# The Spring Course of the Medical Colleges

**Published:** 1884-03

**Authors:** 


### The Spring Course of the Medical Colleges.

For reasons quite obvious to the profession, to which we need not refer in this connection, the medical colleges have arranged for a spring course adapted to both advanced students and beginners in medicine. The importance of Buffalo as an advantageous point to secure both clinical and didactic facilities are thus recognized, and we join in congratulating the profession for this recognition. The announcements have been issued for the Niagara Medical College, as well as for the Buffalo Medical College, and we select the following items of interest to the profession in regard to the new movement.

Niagara Medical College opens its spring course April 3d, and continues ten weeks.

This course is intended to give students an opportunity to pursue their studies in certain directions in a more specific and thorough manner, and to enable them to more carefully and minutely engage in clinical study and observation, and thus to augment that knowledge of disease which is so essential to professional success. It will be arranged so as to accommodate two classes of students; yet there will be no conflict in the hours of teaching, so that each student who may so desire can attend all the lectures and recitations. A *clinical or practical* course of lectures will be given for advanced students and recent graduates, and a *recitation* course will be especially arranged for and adapted to those just entering upon the study of medicine, or who may have attended one term of medical lectures.

### CLINICAL COURSE.

The value and importance of clinical instruction and personal familiarity with medical and surgical diseases require no emphasis in an announcement of this kind. That the ordinary college curriculum and intimate study of disease and methods of treatment, is most forcibly attested in the demand for and success of post-graduate schools in our large medical centres. The Clinical Course in this school is, therefore, inaugurated to supplement the usual didactic teaching which is given, often, to large numbers of students, and which is of a character necessarily limited to the essentials and elementary principles of medicine. It will be as full and complete as the limited time will permit, and superior facilities will be offered by the Faculty for clinical study and observation. The course has been arranged with special reference to the needs of those who are sufficiently advanced to appreciate and understand the application of the principles enunciated in the lecture room, and as exemplified at the bedside and by cases furnished in the hospital wards and dispensary practice, due prominence, however, being given to the most frequent forms of disease and injury with which the young practitioner will meet in the early period of his professional career.

RECITATION COURSE.

In this course the student will pursue the elementary branches of medicine, including Anatomy, Physiology, Chemistry and Materia Medica. The instruction will be largely by recitations from text-books, and by occasional lectures, aided by practical work and demonstrations, and will be suited to young men beginning their medical studies and those who are partially advanced. Able medical gentlemen have been appointed by the Faculty to assist in conducting this course, and every effort will be made to render it of unexceptionable value to the student.

CLINICAL FACILITIES.

The Faculty has at its command the ample facilities of the large and well-equipped Hospital of the Sisters of Charity, the Pine street Emergency Hospital, St. Francis' Hospital, the Washington street Dispensary, the good Samaritan Eye and Ear Infirmary, etc., from which abundant clinical material is furnished for its classes.

DISSECTION.

Abundance of anatomical material is available for students who may wish to dissect, and comfortable apartments will be provided for the purpose.

FACULTY.

*Clinical.*—John Cronyn, M. D., Clinical Medicine. William S. Tremaine, M. D., Clinical and Orthopedic Surgery. Thomas Lothrop, M. D., Clinical

Midwifery. Henry D. Ingraham, M. D., Clinical Gynecology and Pediatrics. William H. Heath, M. D., Clinical Genito-Urinary Diseases. Alvin A. Hubbell M. D.. Clinical Ophthalmology and Otology. Dougald Macniel. M. D., Clinical Dermatology. Floyd S. Crego, M. D., Clinical Nervous Diseases.

*Didactic and Recitation.*—Augustus R. Davidson, M. D., Medical Chemistry. George E. Fell, M. D., Physiology and Microscopy. Charles G. Stockton, M. D., Materia Medica and Medical Botany. Clayton M. Daniels, M. D., Minor Surgery. Frank H. Potter, M. D., Descriptive Anatomy. Herbert Mickle, M. D., Regional and Surgical Anatomy. Frederick R. Campbell, M. D., Hygiene. Carlton C. Frederick, M. D., Physical Diagnosis. Edward Clark, M. D., Emergencies and Pathological Anatomy.

FEES.

The fees for the course are $10.00.

Students who take tickets for the present or the next regular Winter Course will be admitted free.

For further particulars regarding either the Spring or Winter Course of lectures, address the Secretary.

A. A. HUBBELL, M. D

181 Franklin Street, Buffalo, N. Y.

Buffalo Medical College opens its course Monday, March 3d, and continues eight weeks. The following are the faculty and the special departments :

FACULTY.

E. V. Stoddard, M. D., Professor of Materia Medica and Therapeutics; M. D. Mann, M. D., Professor of Obstetrics and Gynæcology; F. W. Abbott, M. D., Lecturer on Otology; A. M. Barker, M. D., Lecturer on Diseases of Children; J. W. Keene, M. D., Lecturer on Obstetrics; Frederick Peterson, M. D., Lecturer on Anatomy; D. F. McPherson, M. D., Lecturer on Minor Surgery; Eli H. Long, M. D., Lecturer on Special Therapeutics; Charles Cary, M. D., Professor of Anatomy; R. A. Witthaus, M. D., Professor of Chemistry; Roswell Park, M. D., Professor of the Principles and Practice of Surgery; Lucien Howe, M. D., Lectur r on Ophthalmology; Bernard Bartow, M. D., Lecturer on Orthopedic Surgery; W. C. Barrett, M. D., Lecturer on Oral Diseases; John H. Pryor, M. D., Lecturer on Physical Diagnosis; Julius Pohlman, M. D., Lecturer on Physiology; Frank A. Vandenburgh, M. D., Lecturer on Chemistry.

The recitation course will be similar in its purposes and advantages to the one above noticed. Clinics will be held at the General

Hospital on Wednesday and Saturday of each week, as heretofore. Dr. Howe will continue his weekly clinics in Ophthalmology.

The "White Library," in the College building, will be open to the students during the day and evening, where books of reference can be consulted, and all the leading medical journals and publications found on file. The Librarian will be in attendance.

An attendance on the Spring Course will be counted as time spent in the study of medicine under the direction of a practitioner, but it cannot be considered as a *course of lectures* in the requirements for graduation.

FEES.

The matriculation fee for this course will be good for the following Winter Term.

General ticket, $15.00.

Special tickets for any single course can be obtained on application to the Secretary. Fee, $5.00.

All communications should be addressed to

J. H. PRYOR, Sec'y and Treas. Spring Faculty,
327 Franklin Street.

